# Patient-experienced effect of an active implementation of a disease management programme for COPD – a randomised trial

**DOI:** 10.1186/1471-2296-14-147

**Published:** 2013-10-03

**Authors:** Margrethe Smidth, Frede Olesen, Morten Fenger-Grøn, Peter Vedsted

**Affiliations:** 1 The Research Unit for General Practice Aarhus, Aarhus University, Aarhus C 8000, Denmark; 2 The Section for General Medicine, Aarhus University, Aarhus C 8000, Denmark

**Keywords:** PACIC, Implementation, Disease management programme, Patient evaluation, COPD, Chronic care model, RCT

## Abstract

**Background:**

People living with chronic disease currently account for the majority of the total healthcare costs. The Central Denmark Region implemented a disease management programme (DMP) for chronic obstructive pulmonary disease (COPD) in 2008. This presented an opportunity to examine the effect of an evidence-based, planned and proactive implementation of a DMP compared to the usual implementation strategy.

**Methods:**

We performed a block- and cluster-randomised controlled trial with two groups and an extra external control group. The primary outcome was patients’ assessment of their care after using an active implementation model for a DMP for COPD measured with the Patient-Assessment-of-Chronic-Illness-Care (PACIC) instrument. At baseline, questionnaires were sent to 2,895 patients identified by an algorithm based on health registry data on lung-related contacts to the healthcare system. Patients were asked to confirm or refute their diagnosis of COPD. Of those who responded, 1,445 (72.8%) confirmed their diagnosis. PACIC data were collected at baseline and at a 12-month follow-up for 744 (51.1%) patients.

**Results:**

Comparing the three groups after the implementation of the DMP, we found a statistically significantly higher change in the PACIC score in the intervention group than in the control groups. No statistically significant differences were found between the control and the external control groups in any of the dimensions.

**Conclusions:**

Reinforcing the role of general practice as coordinator for care-and self-management-support with an active implementation of a DMP for COPD made patients score higher on the PACIC instrument, which indicates a better experience of the received healthcare.

**Trial registration:**

NCT01228708.

## Background

Shared decision-making in healthcare is becoming ever more important. Patients want healthcare professionals to include them in the decisions about their own health, and they want to be involved in the management of their own lives and diseases [1].

The number of patients with chronic disease is growing as a result of inappropriate lifestyle, growing diagnostic activity, improved treatment options and increased life-expectancy [2]. Currently, about a quarter of the 5.6 million Danes [3] are living with one or more chronic diseases, and thus live with multimorbidity.

Patients with multimorbidity often spend much time and encounter many frustrations when they undergo specialised treatment as high-quality multidisciplinary care is often characterized by lack of communication and coordination between health professionals. Patients experience inadequate continuity of care and they face problems in accessing the health professionals they trust [4-6]. Health professionals often consider encounters with patients with multimorbidity demanding and they do not find it easy to delegate responsibility to other professions [7]. The recent rapid growth in technology and specialised treatment makes it feasible to centralise the settings for specialised treatment. Unfortunately, specialised treatment also paves the way for “silo thinking” [8-10], and it is vital that care is tailored to the patients’ needs and that treatment is professional, effective and non-fragmented [11]. People living with chronic diseases currently account for 70-80% of the total healthcare costs [12] and they are more frequently hospitalised than other patients, which is costly and may hamper patient-centred care. A framework for the concerted effort for the care for people living with multimorbidity is needed to give these patients’ an experience of a healthcare system that cooperates to provide the best possible care.

One such strategy is the Chronic Care Model (CCM), which has been successfully implemented in full or partly in different healthcare settings [13,14]. The CCM supports the provision of high-quality care where emphasis is placed on the continuity of care in a strong primary care sector [15] to ensure that patients are given the right care at the right place at the right time and with optimised use of resources [16]; such care should, moreover, be evidence-based, planned and proactive [13].

It becomes important to ensure and support the implementation of such care. This was underpinned in a meta-analysis by Weingarten et al. who concluded that the studied interventions for disease management programmes (DMPs) improved provider adherence and disease control and asked for future studies to directly compare different types of interventions and models for interventions [17]. Patient Assessment of Chronic Illness Care (PACIC) is a patient evaluation tool developed to measures specific actions or qualities of care which patients report that they have experienced in the delivery system [18,19]. Glasgow et al. showed that greater exercise adherence and higher PACIC score [19] were linked for patients with diabetes and a cross-sectional study from the North American insurance company Kaiser Permanente where the CCM is fully implemented concluded that PACIC could be used as a tool for health systems to improve care for chronic diseases [20].

Denmark (3.02 mill citizens who are 35 years old or older) is organised into five regions and 98 municipalities. Responsibility for rehabilitation and preventive services lie with the municipalities, and responsibility for the running of the hospitals and for the provision of service from general practice lies mainly with the regions [21]. Some of the key elements in the CCM model have come to Denmark in the sense that it has become mandatory to jointly plan care in cooperation between the regions and the municipalities healthcare [15,22].

Chronic obstructive pulmonary disease (COPD) is an important chronic disease. Moreover, it is an under-diagnosed, irreversible and potentially life-threatening condition where secondary prevention, treatment and rehabilitation can help control the symptoms, increase the patient’s quality of life and delay disease progression [23]. Newly published results indicate that 14.3% of those who are 35 years or older have COPD [24]; and only approx. 28% of these have been diagnosed [25].

A DMP for COPD was implemented in 2008 in the Central Denmark Region (720,000 citizens aged 35 years old or older) based on the CCM [23]. The programme uses evidence-based clinical and organisational recommendations and is a manual on treatment, task distribution, communication and coordination between stakeholders [12], which has been shown to improve care for chronic conditions [26]. The Central Denmark Region adopted the DMP as standard care for patients with COPD.

To be able to support the patients in assuming an active role in the management of their own illness, we need research-based information, and a recently published cross-sectional study from the Netherlands showed that patients with COPD became more satisfied with their care when a DMP had been implemented [27]. Therefore, we developed an active implementation model to test the active implementation compared to the usual implementation of DMPs [28].

The aim of this paper is to present the effect of the previously developed active implementation model for a DMP for COPD on patients’ assessment of their care.

## Methods

### Study design

The study was a prospective, multicentre, block- and cluster-randomised controlled trial with intervention and control groups and an additional external control group to enable estimates of the extent of spillover effect of the intervention on the local control group [29,30]. The intervention group consisted of patients from a randomly selected half of the general practices in Ringkoebing-Skjern municipality in the Central Denmark Region. Patients from the other half of the practices formed the control group. Patients in a comparable neighbouring municipality all formed the external control group.

**Figure 1:**
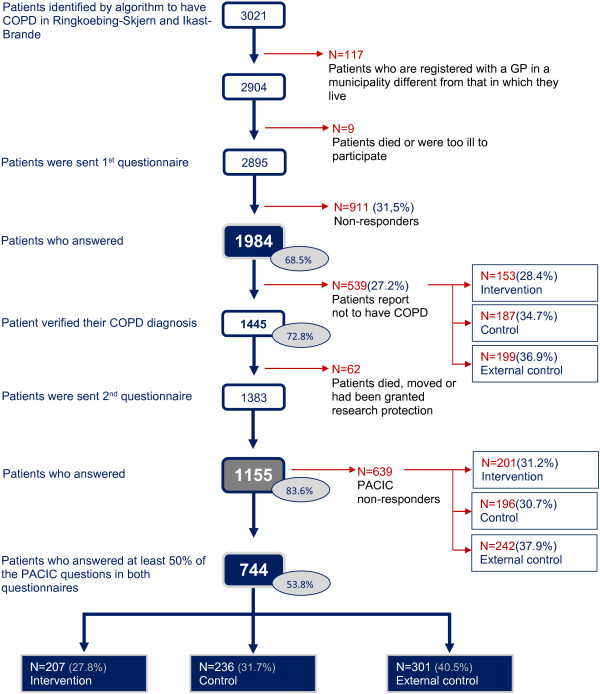
**Flowchart.** Flowchart for inclusion of patients who scored at least 50% of the PACIC instrument in both the baseline and in the follow-up questionnaire a year after the active implementation of a disease management programme for COPD.

A validated COPD algorithm [31] was used to identify the patients who were sent the baseline questionnaire at the start of the intervention. A year later responders to the baseline questionnaire who had confirmed their COPD diagnosis were sent a follow-up questionnaire. Both questionnaires included the PACIC instrument (see Figure 1).

The primary outcome was the patients’ assessment of the care received after implementation of the DMP for COPD based on the CCM [23]. Patients’ assessments were measured with the PACIC instrument [18], which has been developed and validated in the United States to measure the quality of care experienced by the patients as far as the elements of the CCM were concerned; i.e. the community, the healthcare system, self-management support, delivery system design, decision support and clinical information systems. The instrument has been translated and validated within the Danish healthcare system [32].

### Participants

The patient population comprised patients aged 35 years or older from the two municipalities; the patients had to be registered with a GP practice in their residing municipality and identified by the COPD algorithm [31] - where patients were identified from administrative data which indicated whether they had been hospitalised at least once with a lung-related diagnosis during the past five years and/or had redeemed a prescription for lung medication at least twice during the past year and/or had had two spirometries performed at different days during the past year. Furthermore, the patients were the responders to the baseline questionnaire who had confirmed their COPD diagnosis and answered at least 50% of the questions in the PACIC instrument in both questionnaires. The patient population belonged to the group to which their respective GP practices belonged.

The letter of invitation informed the patients about the consequences of their participation and their possibility to withdraw at any time without any consequences for their further treatment within the healthcare system. It was therefore considered equivalent to informed consent to participate in delivering data to the study when patients answered and returned the questionnaire.

### Setting

In Denmark, healthcare is free at the point of care and is tax-financed. The approx. 3,600 Danish GPs have an average of 1,600 patients on their list as approx. 99% of the population is registered with a GP. The GPs are independent contractors with the region and remunerated on a combination of fee-for-service and capitation basis (75/25). GPs act as gatekeepers, and the patients on the list must consult their GP in case of need for medical advice except for emergency room services and for some of the health-related services offered by the municipality.

Ringkoebing-Skjern municipality had approx. 58,000 inhabitants of whom approx. 35,000 were aged 35 or above, and the municipality had 38 GPs organised into 15 practices. The neighbouring municipality (Ikast-Brande) had close to 40,000 inhabitants of whom approx. 24,000 were aged 35 or above, and this municipality had 25 GPs organised into 10 practices. All practices had staff that conducted parts of or the entire consultation on their own. The staff was employed by the GPs and was educated as nurses, laboratory technicians or secretaries.

### Intervention

The intervention practices undertook an active, structured implementation of a DMP for COPD.

The DMP from the Central Denmark Region [23] was based on the GOLD Guidelines [12] and the clinical guideline from The Danish Society for General Practice [33]. It uses evidence-based clinical and organisational recommendations and is a manual on treatment, task distribution, communication and coordination between stakeholders. The programme includes i.a. smoking cessation, yearly follow-up, flu and bronchitis vaccination, advice on comorbidities, diet, exercise and end-of-life care.

The intervention is depicted in the Additional file 1; we have described the development of the intervention in detail elsewhere [28]. The intervention comprised components from the main areas of the CCM - Policies and Resources, Self-Management Support, Delivery System Design, Organisation of Healthcare and Clinical Information System [34]. To stimulate the process, we asked a local, esteemed opinion leader to introduce and support the intervention [35] both to GPs and to the municipality.

The intervention practices were invited to participate in four two-and-a-half-hour sessions. The Breakthrough Series [36] was used as a framework for the implementation of planned and targeted changes. All meetings were chaired by experts and experienced facilitators, who were all clinically educated and experienced in aiding change in practice. One facilitator (MS) visited each practice to explore and/or address challenges encountered in pursuing their goals.

We negotiated our implementation strategy with the municipality, which took active ownership by increasing the number of free COPD courses and smoking cessation courses. The region agreed on providing a special reimbursement to GPs for joint home visits together with the community nurse to newly discharged COPD patients [37].

Targeted self-management support for patients to cope with exacerbations of the disease was an integral part of our strategy, and we developed an action card with advice to patients on management of sputum and exacerbations. The action card was based on the research by Robert Stockley [38].

To provide family, friends and the patients themselves with more knowledge to improve their ability to cope with their disease, we designed a website with information about COPD including contact details to the municipality, patient support groups and the involved GPs.

The standard implementation of the DMP from the Central Denmark Region went ahead and thus also covered all the groups in our study.

### Randomisation and sample size

#### *Randomisation and allocation concealment*

An independent researcher drew slips that were matched to an electronic record with all GP practices in the Ringkoebing-Skjern municipality. The allocation of both GPs and patients was open and known to GPs and researchers for the intervention and the control groups. The patients were not directly informed that their GP practice participated in the study. They simply received the questionnaires with a flyer, and posters were exhibited in the waiting area in the practice. The external control group was only known to the researchers. The practices were block-randomised using three blocks where the first block was solo-practices with two practices randomly allocated to the intervention group and three to the control group. The second block was practices with two GPs: two practices were in the intervention group and three in the control group. The third block was practices with three or more GPs: there were three practices in both the intervention and the control group. There were two solo-practices, three practices with two GPs and four with three or more GPs in the external control group.

Out of the nine invited intervention practices, seven accepted the invitation to participate. One practice with three GPs was allocated to the intervention group as one of the GPs was partly involved in the overall planning of the study. The two practices that declined the invitation were allocated to the control group as we wanted to examine the effectiveness of the intervention. In total, there were 17 GPs in the intervention group, 21 in the control group and 25 in the external control group.

### Sample size

The sample size calculations were based on the primary outcome for the expected changes and α = 0.05, β = 0.1, sampling 1:1 and a minimal, relevant difference of 10%. Approx. 10% of the patients with a chronic disease attended a yearly follow-up consultation; and to ensure statistically power for a change to 20%, we needed to include 360 patients in each of the groups in the randomised controlled trial. For a minimal relevant difference of 10% from 50% to 60%, 1,400 patients needed to be included.

### Patients

The COPD algorithm [31] identified 3,021 patients for inclusion in the study. Of those, 2,895 were sent a baseline questionnaire (see Figure 1). Of the 1,445 patients (72.8% of the responders to the baseline questionnaire) who confirmed their diagnosis, 1,383 (69.7% of the baseline responders) were sent a follow-up questionnaire 12 months later. There were 228 (16.5%) non-responders to the follow-up questionnaire. PACIC scores were collected from the 744 (53.8%) patients who answered at least 50% of the PACIC questions in both the baseline and the follow-up questionnaire.

### Data collection

The PACIC tool includes 20 items in the following five scales: Dimension 1: Patient Activation (items 1–3). Dimension 2: Delivery System Design/Decision Support (items 4–6). Dimension 3: Goal Setting (items 7–11). Dimension 4: Problem-Solving/Contextual Counselling (items 12–15). Dimension 5: Follow-up/Coordination (items 16–20). Each scale is scored by averaging the items completed within that scale, and an overall PACIC score is scored by averaging scores across all 20 items. At least 50% of the items have to be answered to calculate a score. The PACIC was scored by summing participants’ responses across all 20 items. Scores on the PACIC range from 1 to 5 with higher scores indicating a patient’s perception of greater involvement in self-management and receipt of chronic care counselling in line with proactive care provided in line with the CCM.

In their paper on the process of translating and validating the PACIC into Danish, Maindal et al. found that it had good face validity and the internal validation endorsed the five proposed scales [32]. Scores were collected from the patients who had answered at least 50% of all the subscales in the PACIC instrument both at baseline and at follow-up.

### Analyses

To measure the effectiveness of the implementation, we used as-treated analysis, i.e. the practices actually participating in the intervention formed the intervention group. We also performed an intension-to-treat analysis as a sensibility analysis (i.e. the two practices that refused to be in the intervention group were analysed as intervention practices).

We compared the difference between the mean difference in change in scores for the corresponding pairwise comparisons between intervention, control and external control groups to eliminate any variation at baseline. Two-sample *t*-test was used to test the differences because the differences were normally distributed.

At baseline, responders and non-responders were compared in terms of gender and age. Responders with full follow-up and non-responders to the follow-up questionnaire were compared in terms of age and baseline PACIC scores for each scale and the total PACIC score using two-sample *t*-test. The distribution of gender was tested by Pearson's chi-square test.

We applied a significance level of 5%; and in connection with testing of the scales of PACIC, a Bonferroni level of 1% was considered to account for multiple testing. Analyses were performed using STATA version 11.0. (StataCorp, College Station, Texas). The trial followed the consolidated standards of reporting trials guideline extended for cluster-randomised controlled trials [34].

### Ethics

The study was recommended by the Multi Practice Committee of the Danish Society of General Practitioners and the Association of Danish General Practitioners (MPU 17–2009) and approved by the Danish Data Protection Agency (J.nr. 2008-41-2855), the Danish National Board of Health (J. NR.: 7-604-04-2/71/EHE); and the RCT is indexed at http://clinicaltrials.gov/show/NCT01228708. According to the Scientific Ethics Committee for the Region of Central Jutland, the Biomedical Research Ethic Committee System Act did not apply to this project.

## Results

### Baseline characteristics

#### *Patients*

No gender differences between responders and non-responders were observed in the intervention group or in the external control group. The control group counted more men than women (59.7% men (p = 0.007)). No statistically significant age difference between responders and non-responders was found in any of the groups. For the full study population, the responders’ mean age was 67.1 years [95% CI: 66.4;67.9] vs. 66.6 years [95% CI: 65.7;67.6], (p = 0.418) for non-responders (see Table 1).

**Table 1:** Baseline data for patients as listed in the Danish health insurance service registry by 1 November 2008

	**Patients who confirmed that they had COPD**			
	**Intervention**	**Control**	**Ext. control**	**Total**
**N (%)**	424 (29.3)	451 (31.2)	570 (39.4)	1445 (100)
**Men (%)**	206 (48.3)	217 (48.1)	284 (49.8)	707 (48.9)
**Female (%)**	218 (51.7)	234 (51.9)	286 (50.2)	738 (51.1)
**Mean age (min-max)**	68.3 (35–91)	66.5 (35–95)	67.2 (36–95)	67.3 (35–95)
		**Patients who answered at least 50% of the PACIC questions in both questionnaires**			
	**Intervention**	**Control**	**Ext. control**	**Total**	
**n (%)**	207 (27.8)	236 (31.7)	301(40.5)	744 (100)	
**Proportion of N (%)**	48.8	52.3	52.8	51.5	
**Men (%)**	106 (51.2)	126 (53.4)	143 (47.8)	375 (46.6)	
**Female (%)**	101 (48.8)	110 (46.6)	158 (52.2)	369 (53.4)	
**Mean age (min-max)**	68.7 (39–91)	65.8 (35–89)	67.2 (36–90)	67.1 (35–91)	

The difference in baseline score between the patients with follow-up and that of those with no follow-up was only statistically significantly different in the control group in regard to goal setting (dimension 3), where responders with follow-up had a baseline mean score of 1.79 [95% CI: 1.68;1.90] and non-responders one of 1.52 [95% CI:1.34;1.70], (p = 0.013).

### Outcome

Table 2 shows the baseline scores, the follow-up scores and the differences between the groups for PACIC. For all three groups, the mean scores for each of the scales and for the total PACIC score were all below 3, both at baseline and at follow-up (max. score is 5). The scores in the intervention group tended to be higher, though not statistically significantly so, than in both control groups at baseline (see Table 2).

**Table 2:** PACIC scores

**N = 744**	**Intervention**	**Control**	**External control**
	**N = 207**	**N = 236**	**N = 301**
**PACIC dimension 1 – Patient activation**	(203)	(230)	(298)
Baseline	2.39 [2.25;2.54]	2.31 [2.15;2.46]	2.27 [2.15;2.41]
Follow-up	2.48 [2.33;2.64]	2.22 [2.07;2.37]	2.11 [1.99;2.23]
Difference	0.09 [-0.06;0.23]	-0.07 [-0.22;0.08]	-0.16 [-0.28;-0.03]
Difference compared with Control	0.16 [0.05;0.37] p = 0.142	-	-
Difference compared with External control	0.25 [0.05;0.44] p = 0.013	0.09 [-0.10;0.28] p = 0.363	-
**PACIC dimension 2 – Delivery system design/decision support**	(206)	(233)	(299)
Baseline	2.78 [2.65;2.92]	2.76 [2.63;2.90]	2.67 [2.55;2.78]
Follow-up	2.86 [2.73;3.00]	2.63 [2.49;2.77]	2.53 [2.42;2.64]
Difference	0.07 [-0.05;0.19]	-0.12 [-0.25;0.02]	-0.13 [-0.24;-0.02]
Difference compared with Control	0.19 [0.00;0.37] p = 0.044	-	-
Difference compared with External control	0.20 [0.04;0.37] p = 0.018	0.01 [-0.16;0.19] p = 0.868	-
**PACIC dimension 3 – Goal setting**	(204)	(234)	(299)
Baseline	1.74 [1.62;1.89]	1.80 [1.69;1.92]	1.65 [1.55;1.75]
Follow-up	1.88 [1.76;2.00]	1.71 [1.60;1.81]	1.64 [1.55;1.73]
Difference	0.12 [0.01;0.23]	-0.08 [-0.19;0.02]	-0.01 [-0.09;0.08]
Difference compared with Control	0.20 [0.05;0.35] p = 0.009	-	-
Difference compared with External control	0.12 [-0.01;0.26] p = 0.071	-0.08 [-0.21;0.07] p = 0.260	-
**PACIC dimension 4 – Problem-solving/Contextual Counseling**	(204)	(231)	(296)
Baseline	2.26 [2.12;2.40]	2.22 [2.08;2.37]	2.05 [1.93;2.17]
Follow-up	2.33 [2.18;2.48]	2.13 [2.00;2.27]	2.00 [1.89;2.12]
Difference	0.06 [-0.07;0.18]	-0.05 [-0.18;0.08]	-0.06 [-0.16;0.05]
Difference compared with Control	0.11 [-0.08;0.29] p = 0.258	-	-
Difference compared with External control	0.11 [-0.05;0.28] p = 0.172	0.01 [-0.16;0.17] p = 0.929	-
**PACIC dimension 5 – Follow-up/Coordination**	(200)	(223)	(288)
Baseline	1.56 [1.45;1.66]	1.48 [1.40;1.56]	1.44 [1.36;1.51]
Follow-up	1.58 [1.48;1.67]	1.51 [1.42;1.60]	1.42 [1.35;1.49]
Difference	0.01[-0.08;0.11]	0.03 [-0.05;0.12]	-0.05 [0.12;0.03]
Difference compared with Control	-0.02 [-0.15;-0.11] p = 0.760	-	-
Difference compared with External control	0.06 [-0.06;0.18] p = 0.343	0.08 [-0.04;0.20] p = 0.179	-
**PACIC Total – The overall score**	(207)	(236)	(301)
Baseline	2.05 [1.95;2.15]	2.02 [1.92;2.13]	1.92 [1.83;2.01]
Follow-up	2.14 [2.03;2.24]	1.97 [1.86;2.07]	1.87 [1.78;1.95]
Difference	0.08 [0.00;0.16]	-0.05 [-0.14;0.04]	-0.06 [-0.13;0.01]
Difference compared with Control	0.12 [0.00;0.25] p = 0.048	-	-
Difference compared with External control	0.14 [0.03;0.25] p = 0.014	0.01 [-0.10;0.13] p = 0.827	-

The mean PACIC scores for each dimension and the total score for each group recorded at baseline and at follow-up. The change in difference of means scores when comparing the control and external control group, respectively, with the intervention group is shown together with a comparison of change in mean scores between the control and external control groups.

The number of patients who have scored 50% of the dimension is shown for each group in brackets ().

In the intervention group, the total PACIC score rose from 2.06 to 2.14 (difference = 0.08 [95% CI: 0.00;0.16]), while a decrease of 0.05 [95% CI: -0.14;0.04] was seen in the control group. The implied intervention effect was 0.12 [95% CI: 0.00;0.25], (p = 0.048). The effect of the active implementation when comparing the intervention group and the external control group was 0.14 [95% CI: 0.03;0.25], (p = 0.014).

Comparison of the intervention group and the control group showed a change for the scales measuring delivery system design and decision support (dimension 2): 0.19 [95% CI: 0.00;0.37], (p = 0.044) and for the scale of goal setting (dimension 3): 0.20 [95% CI: 0.05;0.35], (p = 0.009).

No difference between the control and the external control group was observed for any of the scales. The only statistically significant difference between men and women was seen in the control group’s goal setting scale (dimension 3), where men scored 1.83 [95% CI: 1.69;1.97] and women 1.60 [95% CI: 1.49;1.72], (p = 0.016).

Although these results were just significant according to the 1% Bonferroni level, together they point in the same direction just like the sensitivity analysis, where the intention-to-treat analysis showed approximately the same patterns as the as-treated analysis. A statistically significant difference in change was observed between the intervention group and the control group on the goal setting scale (dimension 3); the change in the total PACIC score between these two groups was 0.09 [95% CI: -0.03;0.21], (p = 0.134). The overall total PACIC score change between the intervention and the external control group was 0.11 [95% CI: 0.01;0.22], (p = 0.038) (data not shown but available).

## Discussion

In this randomised trial, we found a statistically significant change in the mean differences in the total PACIC score between the intervention group and on the one hand the control group and the external control group on the other hand. The care received was thus given a higher score by patients in the intervention practices than by patients in the control groups.

The results show that the active intervention changed the way patients evaluated their overall care in general and patient activation, delivery system design and decision support and goal setting in particular. However, our results also showed no noteworthy changes for the two dimensions measuring problem-solving/contextual counselling and follow-up/coordination of care. This finding indicates that the implementation of the DMP did not affect the way health professionals support patients in dealing with the challenges of living with COPD and the way they interact with other healthcare providers involved in the patient’s care. It is possible that it is simply too demanding for Danish health professionals to share care considerations both with other healthcare sites and with the patients themselves. This finding highlights a target area for future active implementation models designed to ensure further change in the care for patients living with COPD.

### Comparison with other studies

In a study from the Department of Veterans Affairs in the United States, patients evaluated their care higher if the care they received emphasised self-management, and this meant more for their evaluation than the severity of their disease [39]. In a Danish study where patients assessed the care in general practice using the DANPEP (Danish patients evaluate practice) instrument [40], the patients were more likely to recommend their GP if the care they received was emphatic, patient-oriented, informative and coordinated than if it was easily accessible [41]. In these studies, the patients evaluated their care in one part of the healthcare system only, i.e. general practice. Several randomised controlled trials with complex interventions are being planned in different countries in which the PACIC will be used to measure the effect of the interventions on patients’ assessment of their care from all sectors. One such example is the study by van Lieshout et al. who are planning to measure the effect of two strategies for patients with chronic heart failure using the PACIC [42].

Other studies have used PACIC to illustrate how participation in a DMP affects patients’ evaluation of their care compared to patients who are not in a programme. In one such cluster-randomised study where the researchers administered the PACIC instrument over the telephone 18 months after having implemented a “Guided Care” programme for multi-morbid older persons, the patients had twice as high odds for rating their chronic care highly if they had received the programme than if they had not [43]. The multi-morbid older persons scored higher on all PACIC scales, even when they were not receiving the “Guided Care”, than the intervention patients in our study did after the implementation. The same did patients with COPD in a Dutch study [27] which investigated whether patients enrolled in DMPs perceived the quality of care to be better than those who received the usual care. Compared to these two studies, the intervention patients in our study scored remarkably lower on the Follow-up/Coordination and the Goal setting dimensions, which may point in the direction where the active implementation model would benefit from a stronger focus in the future. Hence, no noteworthy change in these two dimensions was observed in our study.

In a different Dutch study conducted to assess if the care in primary care was congruent with the CCM, patients with cardio-vascular disease and patients with COPD evaluated their care [44]. Again, their scores are higher than the scores for the intervention patients in the present study; and although the pattern for the scores is similar, the greatest difference is for the Follow-up/Coordination and the Goal setting dimensions.

### Strengths and limitations

The strength of our study is its randomised design and the inclusion of an external control group to assess the extent of Hawthorne bias and any spill-over effect. Hawthorne bias could arise if patients were happy that “something” was happening with their care and did not know what else the healthcare system could aspire to provide. This potential bias and any spill-over effect could explain why no statistically significant difference between the intervention group and the control group was found in the patient-activation scale, but the result from the external group makes this less probable.

The Danish version of the PACIC instrument was translated and validated in a population with diabetes and it was culturally adapted [32]. We chose to use the PACIC instrument among others because an Australian study concluded that it was a feasible instrument for comparing patients’ assessment of the quality of care in those situations where they interact with the healthcare system, especially where emphasis is given to self-management [45]. Furthermore, Vrijhoef concluded that the PACIC instrument is the most appropriate instrument among the existing generic instruments that measure patients’ experience of their integrated care for chronic conditions [46].

A weakness of the present intervention, one that is present in most health services research, is that recruitment of patients for the courses covered the whole of Ringkoebing-Skjern municipality and not just the intervention practices. This is one of the obvious drawbacks of health services interventions and this bias would tend to underestimate the actual effect of the intervention.

We chose to only analyse those 53.8% of responders to the follow-up questionnaire who had answered at least 50% of all PACIC questions in both questionnaires. In no way can we reject that these 53.8% of the patients is a selected group who answered at least half instead of some or no PACIC questions at all, and they may not be representative of the full population. We were interested in making a comparison between groups, and the risk for selection connected to randomisation group -“double skewed drop-out” – is therefore considered to be minimal.

There were more men than women in the control group. This could introduce a bias as they might value specific parts of their care in other ways than women. Another bias could be that responders with follow-up scored higher than those without in the control group as far as goal setting was concerned; however, a lower score would only increase the size of the change; and since we compare the mean of the change, the difference would then become even more significant.

The trend towards higher baseline scores for the intervention group could imply that intervention practices already had increased their focus on the collaborative care for the patients with COPD. To assess the effect of the intervention, we used a difference-in-difference approach that captured the change in the mean of the difference between the baseline and the follow-up score whereby we eliminated differences at baseline by focusing only on the change between baseline and follow-up.

Despite our efforts to identify as many of the patients with COPD as possible, we were only able to identify those who had been in contact with the healthcare system where lung-related complaints had caused the healthcare system to take action. It would have been better if the International Coding System for Primary Care (ICPC-coding) had been implemented completely and validated in Danish general practice, which would have made it possible to include also milder degrees of COPD. However, that was not a possibility at the time of this study.

We chose to consider the two GP practices that declined to participate in the intervention as part of the control group because we wanted to examine the effectiveness of the active implementation and they would not be among the practices receiving any of the elements. These two practices were included in our sensibility analysis as intention-to-treat. The difference seen between the intervention group and the control group in the as-treated analysis and the absence of any difference in the intention-to-treat analysis suggests that a possible spill-over effect did not influence the patients’ evaluation.

We could have excluded the two practices that declined invitation. However, if the two practices were just two normal GP practices in Ringkoebing-Skjern, their exclusion would have decreased the statistical precision and this had weakened the conclusions of the randomised study.

## Conclusions

This study showed that patients gave a more positive evaluation of the care they received for their COPD after an active implementation of a DMP for COPD focusing on the GP’s role as a coordinator of the care and on self-management than after standard implementation of a DMP for COPD. Thus, the present study supports the idea of active implementation strategies when implementing new healthcare programmes. The results of the PACIC assessment can also guide us in the direction of where the active implementation model could be improved, namely in the areas of problem solving and follow-up.

## Abbreviations

CI: Confidence Interval; COPD: Chronic Obstructive Pulmonary Disease; DMP: Disease management programme; GP: General Practitioner; PACIC: Patient Assessment of Care for Chronic Conditions.

## Competing interests

The authors declare that they have no competing interests.

## Authors’ contributions

PV and FO participated in the design of the study and the drafting the manuscript. MFG assisted with statistical analysis and drafting of the manuscript. MS participated in the design and coordinated the study while drafting the manuscript. All authors have read and approved the final manuscript.

## Pre-publication history

The pre-publication history for this paper can be accessed here:

http://www.biomedcentral.com/1471-2296/14/147/prepub

## Supplementary Material

Additional file 1 **The PaTPlot depicting the timeline and the contents of the active implementation model.** Squares illustrate fixed objects, e.g. printed materials like questionnaires. Circles illustrate that an activity was involved in the component, e.g. Continued Medical Education meetings. Click here for file
