# Supplementary material for: Patient-experienced effect of an active implementation of a disease management programme for COPD – a randomised trial
Source: BMC Fam Pract. 2013 Oct 3;14:147. doi: 10.1186/1471-2296-14-147 (PMC3850694; doi:10.1186/1471-2296-14-147)
Supplement: Additional file 1 — The PaTPlot depicting the timeline and the contents of the active implementation model. Squares illustrate fixed objects, e.g. printed materials like questionnaires. Circles illustrate that an activity was involved in the component, e.g. Continued Medical Education meetings. [file 1471-2296-14-147-S1.doc]

| **TIMELINE** | **INTERVENTION** | **CONTROL** | **EXTERNAL CONTROL** |
| --- | --- | --- | --- |
| -3 weeks | 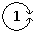 | 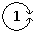 |  |
| Week 1 | Patients identified by COPD algorithm | | |
|  | General practices block-randomized | | No randomization |
| Week 2 | 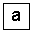 | 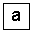 |  |
| Week 3 | 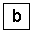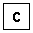 | 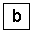 | 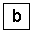 |
| Week 4 | 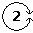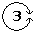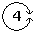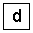 |  |  |
| Week 5 | 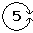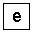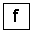 |  |  |
| Week 9 | 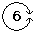 |  |  |
| Week 11 | 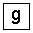 |  |  |
| Week 12 | 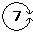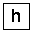 |  |  |
| Week 12-16 | 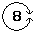 |  |  |
| Week 22 | 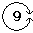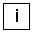 |  |  |
| Week 24 | 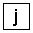 |  |  |
| Week 28 | 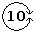 |  |  |
| Week 38 | 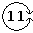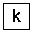 |  |  |
| Week 40 | 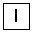 |  |  |
| Week 44 | 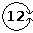 |  |  |
| Week 50 | 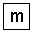 |  |  |
| Week 52 | 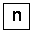 | 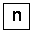 | 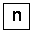 |
| Week 54 | 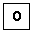 | 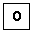 |  |
| Week 110 | 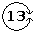 | 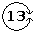 |  |

| 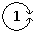 | 15 general practices invited to a meeting with information about the study |
| --- | --- |
| 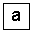 | Baseline questionnaires sent to general practitioners. http://kol.au.dk/fileadmin/www.kol.au.dk//mest_til_praksis/sp__rgeskemaunders__gelse/sp.pdf |
| 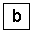 | Baseline questionnaires including the PACIC instrument sent to patients identified by the COPD algorithm. http://kol.au.dk/fileadmin/www.kol.au.dk/mest_til_patienter/spunder/patientskema.pdf |
| 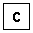 | Information about project and places to go for information about COPD supplied in a flyer sent with the questionnaire. |
| 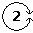 | Project head developed and implemented feedback paper from health centre to general practitioners when patients had finished "Stop smoking" or attended COPD courses together with the staff. The health centre increased the course capacity. |
| 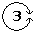 | Project head held meetings with staff and the COPD consultant at the three hospitals. General practices received a fax from the hospital when one of their patients with COPD had been discharged. |
| 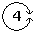 | Project head visited all general practices. Options and possibilities for each clinic to change treatment and procedures for patients with COPD were discussed. |
| 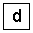 | Referral papers to "Stop smoking" and "Living with COPD" courses at the health centre sent to each general practice. |
| 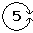 | 1. Breakthrough meeting with general practitioners and clinic staff. Consultant specialized in COPD updated on evidence-based care. Specialist in implementing change in practice introduced the Breakthrough Series and shared care in general practice. Health centre staff introduced the "Living with COPD" course and told about its contents to the general practices. |
| 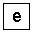 | Each general practice supplied with a list of the COPD population identified by the COPD algorithm in their clinic. |
| 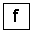 | Action card and explanatory brochure distributed to general practices with verbal information on how to inform patient about the use of the action card. http://kol.au.dk/menu1/dksgn/handlingskort/ |
| 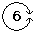 | Project head called to the project coordinator at each general practice to exchange experience and to encourage each practice to keep implementing small changes with the Breakthrough Series. |
| 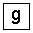 | http:\\kol.au.dk. Website launched with information for patients and general practices. Advertised to general practices and the health centre by email. |
| 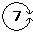 | 2. Breakthrough meeting with general practitioners and staff. General practitioners and practice staff work discussed which of the changes worked in their practices and which needed to be adjusted. Each practice developed plan for change to be implemented before next meeting. |
| 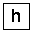 | Poster for display in the waiting room of the general practices advertising the website supplied to each practice at Breakthrough meeting. |
| 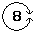 | Project head visited the general practices that wanted to exchange views on their progress and to get support. |
| 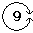 | 3. Breakthrough meeting with general practitioners and practice staff. Professional patient tells her story of living with COPD and her experience of the healthcare system. Practice consultant tells how he supports patients' self-management. Some baseline data are presented from the patient’s questionnaire. |
| 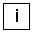 | Each person wrote a postcard to themselves reminding them what two things they wanted to see implemented in their practice during the next half a year. |
| 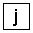 | Film clip on: "A smoker wanting to give up" consultation - "How to do a spirometry" - "A 12-month check-up for a patient with COPD". Film clips are on the website to remind general practitioners and practice staff of all components prescribed in the disease management program. http:\\kol.au.dk. |
| 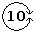 | Project head visited the hospitals to encourage continued practice of sending a fax to general practice when one of their patients with COPD was discharged. |
| 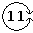 | Project head visited general practices to encourage continued focus on the program’s recommendations for treatment and care of patients with COPD. |
| 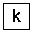 | Practice staff is sent the postcard reminding them of which two things they wanted to see implemented in their practice. |
| 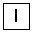 | More action cards and brochures sent to the general practices to maintain focus on support for self-management. |
| 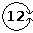 | Project head visited general practices to encourage continued focus on the program’s recommendations for treatment and care of patients with COPD. |
| 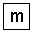 | General practitioners were sent the postcard reminding them of which two things they wanted to have implemented in their practice. |
| 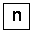 | Patients who confirmed their diagnosis of COPD in the baseline questionnaire were sent a follow-up questionnaire including the PACIC instrument. http://kol.au.dk/fileadmin/www.kol.au.dk/mest_til_patienter/spunder/2010_Spoergeskema_hjemmesiden.pdf |
| 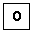 | The general practices, the health centre and the hospitals were informed by email of the collection of register data for outcome measures for the study. |
| 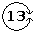 | General practices, health centre and COPD consultant were invited to a meeting with presentation of the results from the study. |
